# EXPLORING MULTIDIMENSIONAL RELATIONSHIPS OF OLDER ADULTS LIVING WITH CHRONIC PAIN

**DOI:** 10.1093/geroni/igad104.3330

**Published:** 2023-12-21

**Authors:** Taylor Munson, Megan Curtis, Jennifer Brailsford, Phyllis Hendry, Sophia Sheikh

**Affiliations:** University of Florida College of Medicine Jacksonville, Jacksonville, Florida, United States; University of Florida College of Medicine Jacksonville, Jacksonville, Florida, United States; University of Florida, Gainesville, Florida, United States; University of Florida College of Medicine Jacksonville, Jacksonville, Florida, United States; University of Florida College of Medicine Jacksonville, Jacksonville, Florida, United States

## Abstract

Older adults who experience chronic pain (CP) face many challenges from their illness, which can affect their personal and healthcare relationships. Research indicates the importance of relationships on health and quality of life outcomes in older adults with CP. However, minimal research has explored their perspectives on the relationships they have with their healthcare providers, healthcare system, their CP, and family/friends/community. Objective: To understand how older adults’ CP impacts their relationship with the healthcare system; and how older adults’ CP impacts their personal relationships (i.e. self, family, community).

Methods: Adults >50 years of age with pain were eligible to enroll in virtual focus groups and/or interviews. Audio recordings were transcribed, coded, and analyzed via a mixed inductive-deductive framework approach using ATLAS.ti. Microsoft. Descriptive statistics were performed using Stata 16.

Results: Sixteen participants participated in a focus group (n=3) or an interview (n=13). Participants identified as White (n=12,75%), female (n=12,75%), and were between the ages of 55-64 years old (n=8,50%). Most participants held an associate’s degree or higher (n=15,93%) and reported private medical insurance coverage (n=11,70%). Sessions lasted between 30-90 minutes. Participants described complex relationships that could have positive or negative impacts on their ability to manage their CP and their overall quality of life. Relationships explored included the healthcare system, clinician(s), family, other patients, and CP.

Discussion: The relationships of older adults living with CP are multidimensional and can support or adversely affect pain management, mental health, and quality of life.

